# A real-world investigation of drug-induced hyperglycemia and diabetes mellitus in pediatric populations using the FDA adverse event reporting system

**DOI:** 10.3389/fmed.2026.1751155

**Published:** 2026-01-28

**Authors:** Xue Zhao, Xiutian Sun, Meng Ma, Lijuan Teng, Xiaohua Qu

**Affiliations:** Weifang People’s Hospital, Shandong Second Medical University, Weifang, Shandong, China

**Keywords:** drug-induced diabetes mellitus, drug-induced hyperglycemia, FAERS, pediatric populations, pharmacovigilance

## Abstract

**Background:**

Pediatric drug-induced hyperglycemia/diabetes mellitus (DIH/DIDM) is regarded as a preventable iatrogenic condition; nevertheless, its clinical under-recognition persists, and age-specific pharmacovigilance evidence remains critically deficient. This study conducted a retrospective pharmacovigilance investigation to identify risk signals associated with pediatric DIH/DIDM.

**Research design and methods:**

We extracted pediatric (age < 18 years) adverse-event reports from the FDA Adverse Event Reporting System (2004 Q1–2025 Q1) and retained those mapped to any of nine predefined MedDRA Preferred Terms (PTs) for hyperglycemia or diabetes mellitus. A disproportionality analysis was subsequently performed to estimate the reporting association between suspect drugs and the events of interest.

**Results:**

We mined 504,458 pediatric adverse-event reports from the U.S. FDA Adverse Event Reporting System (2004Q1–2025Q1) and detected 195 positive drug–adverse event signals. Among 2,436 pediatric DIH/DIDM cases, 72% were adolescents aged 10–18 years; the overall median age was 13 years (IQR 9–16). Antineoplastic agents, glucocorticoids, immunosuppressants, psychotropic medications, and growth hormone constituted five cardinal risk clusters, and Off-label signals for drugs—including minocycline and montelukast—were captured for the first time. Underlying conditions were predominantly acute lymphoblastic leukemia, neuropsychiatric disorders, immunosuppression-related disorders, and growth hormone deficiency.

**Conclusion:**

Our study systematically delineated the risk signals implicated in pediatric DIH/DIDM. Clinicians should heighten surveillance when prescribing potentially diabetogenic agents and pay particular attention to age-related windows of susceptibility.

## 1 Introduction

The worldwide surge in pediatric diabetes mellitus (DM) has emerged as a critical public health concern (1–3). In the United States, SEARCH 2002–2018 shows type 1 diabetes incidence in 0–19-year-olds rising to 22.2 per 100000 person-years in 2017/2018 (+ 2.0% per year), while type 2 diabetes in 10–19-year-olds reached 17.9 per 100 000 (+ 5.3% per year) (1). While T1D remains the predominant form in childhood, T2D and secondary forms—particularly drug-induced diabetes mellitus (DIDM)—are emerging contributors (4). Against the backdrop of these concurrent epidemics of pediatric diabetes, DIDM has evolved into an insidious yet potentially preventable etiological factor. A contemporary Canadian prospective surveillance program (2017–2019) documented 0.44 medication-induced pediatric cases per 100 000 children per year, with glucocorticoids implicated in 96% of events-60% as monotherapy and 40% in combination. Mean age at diagnosis was 13.5 years; 29% of affected children were already obese, and 58% were asymptomatic, underscoring the risk of delayed recognition (5). Tosur et al. provided robust evidence that glucocorticoids, l-asparaginase, tacrolimus, sirolimus, and second-generation antipsychotics (e.g., risperidone and olanzapine) reproducibly precipitate hyperglycemia in children, with a subset of patients progressing to diabetic ketoacidosis. By contrast, reports linking basiliximab, nivolumab, pembrolizumab, and thiazide diuretics to diabetes mellitus originate predominantly from adult cohorts; large-scale pediatric pharmacovigilance studies are still required to quantify their glycemic impact in childhood. Independent of drug class, cumulative dose and patient-specific susceptibility traits—most prominently obesity and a positive family history of diabetes—synergistically heighten the risk of hyperglycemia in the pediatric population (6). Drug-induced hyperglycemia/diabetes mellitus (DIH/DIDM) in children is generally reversible: glucose levels normalize within days to weeks after dose reduction or withdrawal. A 13-year-old child with nephrotic syndrome presented with diabetes while on prednisone plus tacrolimus; rapid tapering of prednisone promptly decreased insulin requirements (7). An 11-month-old infant receiving high-dose sirolimus developed hyperglycemia after 17 days. cessation of sirolimus and 14 days of transient insulin therapy restored normoglycemia (8), confirming the reversible nature of DIH/DIDM.

With the increasing diversity of clinical therapeutic drugs, the scope of agents linked to blood glucose dysregulation adverse events is constantly expanding, driving the continuous evolution of the overall characteristics of drug-associated metabolic risks in children. Second-generation antipsychotics, checkpoint inhibitors, calcineurin inhibitors and asparaginase are now routinely prescribed in pediatric oncology, transplant and psychiatric indications (6, 9). These agents disrupt glucose homeostasis via β-cell apoptosis, peripheral insulin resistance, augmented hepatic gluconeogenesis, and incretin-pathway modulation (9–11). Developmental pharmacology further magnifies vulnerability: lower hepatic clearance, immature counter-regulatory hormone networks and transient pubertal insulin resistance collectively amplify drug exposure and metabolic perturbation (12). Despite these converging mechanisms, current risk estimates derive almost exclusively from adult pharmaco-epidemiology or sporadic pediatric case reports (6, 13), leaving clinicians to extrapolate without age-specific dosing, off-label use or growth-related confounders being considered.

To redress the acute shortage of pediatric-specific evidence, we extracted the entire FAERS public-release corpus and, after rigorous deduplication and terminology standardization, performed what is to our knowledge the first disproportionality analysis confined to drug-associated hyperglycemia/diabetes mellitus in 0–18-year-olds. By combining experience reporting rates with well-defined glucose dysregulation mechanisms, we attempted to identify pharmacological entities with the highest excess risk and their treatment indications, and analyze the differences in incidence among children of different age groups. The resultant safety signals are intended to equip pediatric prescribers with evidence-grounded surveillance metrics, thereby enabling prospective, point-of-care risk mitigation.

## 2 Materials and methods

### 2.1 Data acquisition

Adverse event (AE) records were retrieved from the U.S. Food and Drug Administration Adverse Event Reporting System (FAERS) quarterly releases covering 85 consecutive quarters (2004 Q1–2025 Q1), accessible via the public repository at https://fis.fda.gov/extensions/FPD-QDE-FAERS/FPD-QDE-FAERS.html.

### 2.2 Data processing procedure

After establishing a local PostgreSQL instance, raw FAERS files were parsed and harmonized with Python-based ETL scripts. Drug identifiers were mapped to the RxNorm clinical vocabulary, and event descriptors and indications were aligned with MedDRA v28.0 preferred terms (PTs). In the FAERS database, drugs are categorized into four roles: Primary Suspect Drug, Secondary Suspect Drug, Concomitant Drug, and Interacting Drug. Eligible reports were restricted to pediatric subjects (age 0–18 years) where the suspect drugs were flagged as “Primary Suspect (PS)”and “Secondary Suspect (SS).” Events consistent with drug-induced hyperglycemia/diabetes mellitus (DIH/DIDM) were identified using the PTs “Type 1 diabetes mellitus,” “Type 2 diabetes mellitus,” “Diabetes mellitus,” “Fulminant type 1 diabetes mellitus,” “Hyperglycemia,” “Hyperglycemic crisis,” “Glucose tolerance impaired,” “Impaired insulin secretion,” and “Diabetic ketoacidosis.” The method for removing duplicate reports is as follows: select the PRIMARYID, CASEID and FDA_DT fields from the DEMO table, sort them by CASEID, FDA_DT and PRIMARYID in sequence; for reports with the same CASEID, retain the one with the maximum FDA_DT value; for reports with identical CASEID and FDA_DT values, retain the one with the maximum PRIMARYID value.

### 2.3 AE signal detection and analysis

Disproportionality was quantified with the reporting odds ratio (ROR = ad/bc) and its 95% CI [lnROR ± 1.96 √(1/a + 1/b + 1/c + 1/d)]. In the 2 × 2 contingency framework, a denotes reports listing both the drug of interest and the target diabetes-related ADR; b denotes those listing the same drug but any other ADR; c denotes those listing the target ADR but attributed to any other drug; and d denotes reports listing neither the drug nor the target ADR. A signal was considered positive when the lower 95% CI boundary exceeded unity and the case count (a) was ≥ 3. All computations were performed in R software (version 4.1.0). To delineate age-related risk patterns, subjects were partitioned into four strata (0–4, 5–9, 10–14, and 15–18 years), and stratum-specific suspect agents were isolated.

## 3 Results

### 3.1 Descriptive analysis

**FIGURE 1:**
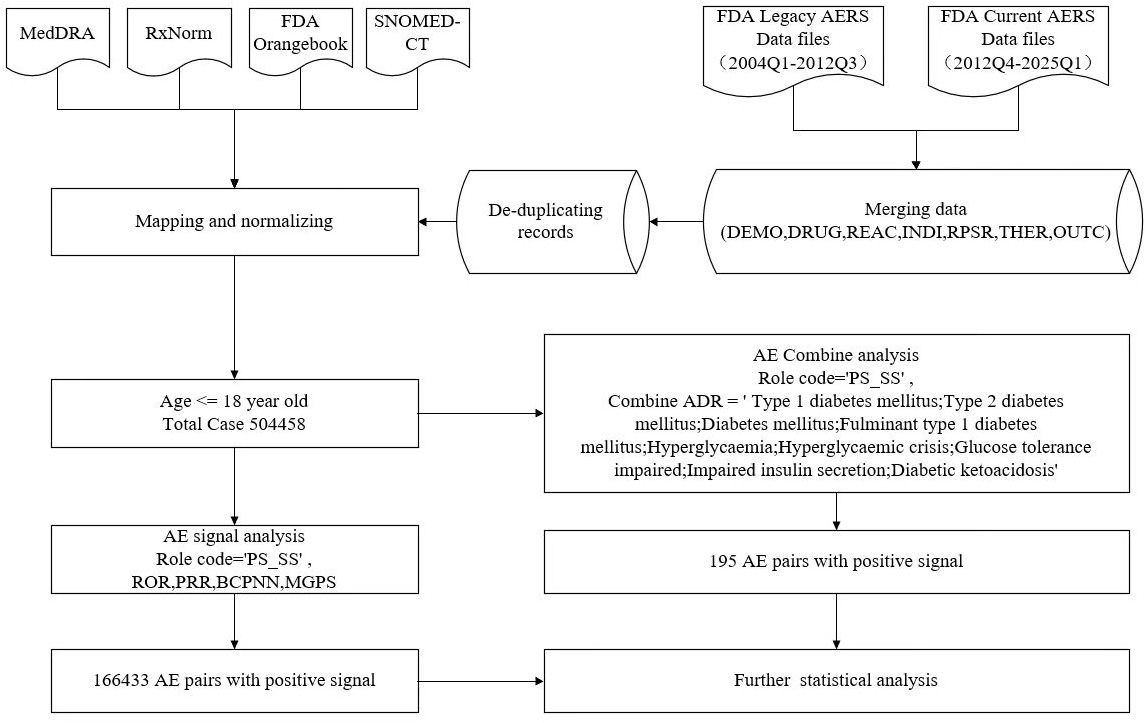
Flow diagram of data extraction and cleaning.

**FIGURE 2:**
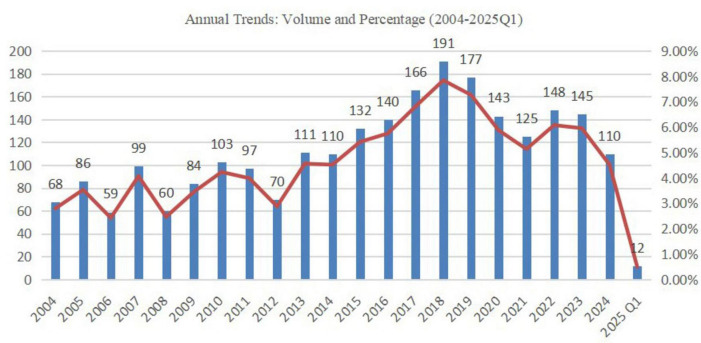
Trend graph of annually reported adverse event numbers for drug-induced hyperglycemia/diabetes mellitus from 2004 to Q1 of 2025.

From Q1 2004 to Q1 2025, we analyzed 504,458 pediatric AE cases. The Prisma diagram is shown in Figure 1. AEs of PS/SS identified through PT screening yielded 2,436 hyperglycemia/diabetes-associated cases. Disproportionality analysis revealed 195 positive drug–adverse event signals. Analysis of 2,436 pediatric DIH/DIDM cases retrieved from FAERS is summarized in Table 1. After excluding 53 records (2.18%) with missing gender data, the analyzed cohort comprised 1,237 males (50.78%) and 1,146 females (47.04%). Notably, females predominated over males in the 10–14-year stratum (51.33% vs. 48.67%). Age distribution peaked in the 10–14-year group (902 cases, 37.03%), followed by the 15–18-year group (852 cases, 34.98%), the 5–9-year group (370 cases, 15.19%), and the 0–4-year group (312 cases, 12.81%). The overall mean age was 11.94 ± 5.03 years, with a median of 13 years (IQR 9–16). Physicians filed most reports (894 cases, 36.70%), followed by other health professionals (848 cases, 34.81%). Geographically, the United States submitted the majority (1,128 cases, 46.31%), followed by Japan (196 cases, 8.05%) and Canada (150 cases, 6.16%). Regarding clinical outcomes, “Other Serious (Important Medical Event)” was most common (1,576 cases, 46.54%), followed by “Hospitalization—Initial or Prolonged” (1,223 cases, 36.12%), with 174 death cases (5.14%). Annual trend data (Figure 2) showed fluctuating reporting numbers, peaking in 2018 (191 cases) followed by 2019 (177 cases).

**TABLE 1:** Patient characteristics in pediatric drug-induced hyperglycemia/diabetes mellitus.

Demographic variables		Cases, n (%)			
		Total	Female	Male	Missing
Age group (year)	0–18	2436	1146(47.04)	1237(50.78)	53(2.18)
	0–4	312(12.81)	126(40.38)	173(55.45)	13(4.17)
	5–9	370(15.19)	166(44.86)	195(52.7)	9(2.43)
	10–14	902(37.03)	463(51.33)	423(46.90)	16(1.77)
	15–18	852(34.98)	391(45.89)	446(52.34)	15(1.76)
Age (year)	Mean (SD)	11.94(5.03)			
	Median (Q1, Q3)	13(9.16)			
Reporters’ occupations	Consumer	382(15.68)			
	Lawyer	59(2.42)			
	Other health professional	848(34.81)			
	Pharmacist	67(2.75)			
	Physician	894(36.70)			
	Missing	182(7.47)			
	Other	4(0.16)			
Reporting country	United States	1128(46.31)			
	Japan	196(8.05)			
	Canada	150(6.16)			
	France	110(4.52)			
	United Kingdom	91(3.74)			
	Missing	118(4.84)			
	Others	643(26.40)			
Outcome	Death	174(5.14)			
	Life-threatening	312(9.21)			
	Hospitalization—initial or prolonged	1223(36.12)			
	Disability	69(2.04)			
	Congenital anomaly	4(0.12)			
	Required intervention to prevent permanent impairment/damage	28(0.83)			
	Other serious (important medical event)	1576(46.54)			

### 3.2 Disproportionality analysis

Using the Reporting Odds Ratio (ROR) method, we systematically evaluated drug safety signals associated with pediatric DIH/DIDM (Table 2). Disproportionality analysis of 2,436 pediatric DIH/DIDM cases (nine MedDRA PTs) revealed somatropin (302 cases; ROR 2.87, 95% CI: 2.55–3.23), tacrolimus (265 cases; ROR 4.86, 95% CI 4.28–5.52), and vincristine (265 cases; ROR 2.79, 95% CI 2.46–3.17) were the most frequently implicated agents. Corticosteroids exhibited a pronounced, stepwise risk gradient: prednisone (238 cases; ROR 5.12, 95% CI 4.48–5.86), dexamethasone (232 cases; ROR 4.72, 95% CI 4.12–5.41), prednisolone (173 cases; ROR 4.62, 95% CI 3.96–5.04), and methylprednisolone (158 cases; ROR 4.28, 95% CI 3.64–5.04). Additional high-risk antineoplastics included pegaspargase (186 cases; ROR 5.80, 95% CI 4.99–6.74), methotrexate (258 cases; ROR 2.02, 95% CI 1.78–2.29), cytarabine (164 cases; ROR 2.61, 95% CI 2.23–3.06), and cyclophosphamide (122 cases; ROR 1.36, 95% CI 1.13–1.63). Among second-generation antipsychotics, quetiapine (195 cases; ROR 6.15, 95% CI 5.30–7.12), risperidone (183 cases; ROR 2.91, 95% CI 2.50–3.38), and olanzapine (133 cases; ROR 6.65, 95% CI 5.57–7.94) generated significant signals.

**TABLE 2:** Top 15 pediatric drugs with disproportionate hyperglycemia/diabetes reports.

Drug-induced hyperglycemia and diabetes mellitus			Drug-induced type 1 diabetes mellitus			Drug-induced type 2 diabetes mellitus			Drug-induced diabetic ketoacidosis		
Drug name	Number of cases	ROR (95% CI)	Drug name	Number of cases	ROR (95% CI)	Drug name	Number of cases	ROR (95% CI)	Drug name	Number of cases	ROR (95% CI)
Somatropin	302	2.87(2.55–3.23)	Somatropin	89	5.57(4.43–7.01)	Quetiapine	57	30.86(22.84–41.69)	Tacrolimus	78	12.31(9.62–15.77)
Tacrolimus	265	4.86(4.28–5.52)	Quetiapine	46	8.87(6.54–12.01)	Somatropin	33	4.26(2.95–6.15)	Quetiapine	43	10.73(7.81–14.73)
Vincristine	265	2.79(2.46–3.17)	Olanzapine	30	9.03(6.24–13.07)	Vincristine	25	3.76(2.48–5.70)	Somatropin	36	2.60(1.84–3.66)
Methotrexate	258	2.02(1.78–2.29)	Aripiprazole	28	4.33(2.95–6.34)	Aripiprazole	23	8.62(5.59–13.27)	Prednisone	31	5.05(3.50–7.29)
Prednisone	238	5.12(4.48–5.86)	Isotretinoin	27	2.69(1.83–3.97)	Risperidone	23	5.31(3.45–8.17)	Olanzapine	21	7.96(5.13–12.37)
Dexametasone	232	4.72(4.12–5.41)	Minocycline	25	20.37(13.58–30.56)	Lomustine	19	329.69(200.32–542.59)	Metformin	21	17.46(11.22–27.16)
Quetiapine	195	6.15(5.30–7.12)	Prednisolone	23	3.62(2.38–5.51)	Levetiracetam	19	6.36(3.97–10.18)	Asparaginase *Escherichia coli*	18	7.69(4.79–12.36)
Pegaspargase	186	5.80(4.99–6.74)	Tacrolimus	23	2.41(1.58–3.66)	Carboplatin	19	11.85(7.40–18.97)	Prednisolone	17	3.39(2.09–5.52)
Risperidone	183	2.91(2.50–3.38)	Risperidone	22	2.07(1.35–3.17)	Tioguanine	19	33.79(21.06–54.21)	Canagliflozin	17	676.15(372.41–1227.65)
Prednisolone	173	4.62(3.96–5.40)	Dexametasone	16	1.85(1.13–3.05)	Procarbazine	19	196.64(120.84–319.99)	Dexametasone	16	2.38(1.44–3.92)
Cytarabine	164	2.61(2.23–3.06)	Montelukast	13	1.78(1.03–3.10)	Vinblastine	19	86.40(53.61–139.25)	Aripiprazole	15	2.89(1.72–4.84)
Methylprednisolone	158	4.28(3.64–5.04)	Asparaginase *Escherichia coli*	13	4.28(2.46–7.43)	Triptorelin	18	84.00(51.51–136.99)	Pegaspargase*	13	2.96(1.71–5.15)
Olanzapine	133	6.65(5.57–7.94)	Valproic acid	13	1.86(1.07–3.22)	Hydrocortisone	17	11.59(7.07–19.02)	Raltegravir	9	17.17(8.83–33.39)
Cyclophosphamide	122	1.36(1.13–1.63)	Everolimus	9	4.82(2.49–9.34)	Olanzapine	16	11.20(6.73–18.64)	Daunorubicin	8	2.56(1.27–5.15)
Aripiprazole	111	2.79(2.30–3.38)	Hydrocortisone	8	2.21(1.10–4.47)	Prednisone	12	3.45(1.93–6.18)	Dapagliflozin	7	135.77(61.85–298.06)

*Pegaspargase is the PEGylated form of Asparaginase *Escherichia coli*.

Analysis of drug-induced type 1 diabetes mellitus identified growth hormone preparations as particularly noteworthy: somatropin (89 cases, ROR = 5.57, 95%CI: 4.43–7.01). Atypical antipsychotics displayed especially strong associations, with olanzapine (30 cases, ROR = 9.03, 95%CI: 6.24–13.07) and quetiapine (46 cases, ROR = 8.87, 95%CI: 6.54–12.01) showing prominent signals. Notably, minocycline demonstrated unexpectedly elevated risk (25 cases, ROR = 20.37, 95%CI: 13.58–30.56).

For drug-induced type 2 diabetes mellitus, lomustine (19 cases, ROR = 329.69, 95%CI: 200.32–542.59) and procarbazine (19 cases, ROR = 196.64, 95%CI: 120.84–319.99) showed pronounced signals. Quetiapine maintained significant association (57 cases, ROR = 30.86, 95%CI: 22.84–41.69), while somatropin (33 cases, ROR = 4.2, 95%CI: 2.95–6.15), vincristine (25 cases, ROR = 3.76, 95%CI: 2.48–5.70), and risperidone (23 cases, ROR = 5.31, 95%CI: 3.45–8.17) exhibited distinctive risk patterns.

The most pronounced signals in drug-induced diabetic ketoacidosis were observed with SGLT-2 inhibitors: canagliflozin exhibited an extreme disproportionality (17 cases; ROR 676.15; 95% CI: 372.41–1227.65), followed by metformin (21 cases; ROR 17.46; 95% CI: 11.22–27.16). Among immunosuppressants, tacrolimus carried the highest risk (78 cases; ROR 12.31; 95% CI: 9.62–15.77). Significant associations were also detected for the antipsychotics quetiapine (43 cases; ROR 10.73; 95% CI: 7.81–14.73) and olanzapine (21 cases; ROR 17.46; 95% CI: 11.22–27.16), as well as for the integrase inhibitor raltegravir (9 cases; ROR 17.17; 95% CI: 8.83–33.39). Somatropin was observed with a high number of reports (36 cases; ROR 2.6; 95% CI: 1.84–3.66).

In the assessment of hyperglycemia/diabetes-related adverse events, the prescribing information for vincristine, methotrexate, and daunorubicin did not mention such reactions. Among the 15 drugs associated with type 1 diabetes in Table 2, minocycline, and montelukast did not mention hyperglycemia or type 1 diabetes, while other drugs in this group only mentioned hyperglycemia without specifically referring to type 1 diabetes. Among the 15 drugs associated with type 2 diabetes in Table 2, lomustine, levetiracetam, carboplatinpro, carbazine, and procarbazine did not mention hyperglycemia-related adverse events, and other drugs in this group similarly only mentioned hyperglycemia without specifically referring to type 2 diabetes. Regarding diabetic ketoacidosis (DKA), olanzapine, canagliflozin, dapagliflozin, and empagliflozin explicitly listed DKA as an adverse reaction, while metformin mentioned DKA under contraindications. No other drugs included content related to DKA.

### 3.3 Age-stratified drug exposure

The age-stratified analysis revealed significant differences in drug exposure patterns (Table 3). The 0–4 year group showed predominant use of chemotherapeutic agents (vincristine 33 cases, methotrexate 34 cases) along with glucocorticoids such as dexamethasone (30 cases). In the 5–9 year group, dexamethasone use increased markedly (68 cases), while growth hormone therapy (somatropin 55 cases) and psychotropic medications (risperidone 43 cases) began to emerge. The 10–14 year group demonstrated overlapping drug risks, including peak growth hormone administration (somatropin, 175 cases) and glucocorticoids such as prednisone (104 cases), alongside sharply rising immunosuppressants like tacrolimus (96 cases) and psychotropic drug use (quetiapine 92 cases). The 15–18 year group exhibited concurrent peaks in both immunosuppressant use (tacrolimus 132 cases) and psychotropic medications (quetiapine 81 cases, olanzapine 76 cases), while maintaining high glucocorticoid exposure (prednisone 95 cases).

**TABLE 3:** Top 15 medications by reported cases across age groups.

Drug name	Total	0–4 year	5–9 year	10–14 year	15–18 year
Somatropin	302	8	55	175	64
Vincristine	265	33	59	96	77
Tacrolimus	265	12	25	96	132
Methotrexate	258	34	56	95	73
Prednisone	238	24	15	104	95
Dexametasone	232	30	68	82	52
Quetiapine	195	1	21	92	81
Pegaspargase	186	24	28	73	61
Risperidone	183	5	43	74	61
Prednisolone	173	14	23	77	59
Cytarabine	164	23	28	63	50
Methylprednisolone	158	18	23	58	59
Olanzapine	133	4	15	38	76
Cyclophosphamide	122	11	19	43	49
Aripiprazole	111	3	14	52	42

### 3.4 Indications of drug-hyperglycemia/diabetes

After excluding records with missing data, Table 4 presents the putative indications for pediatric cases of drug-induced hyperglycemia/diabetes mellitus reported in FAERS. Acute lymphoblastic leukemia (ALL, 192 cases, 21.3%) was the predominant indication, closely linked to glucocorticoid and chemotherapeutic regimens. Neuropsychiatric disorders collectively accounted for substantial proportions, led by bipolar disorder (111 cases), attention-deficit/hyperactivity disorder (83 cases), autism spectrum disorder (71 cases), and depression (69 cases). Endocrine and immune-related conditions, including growth hormone deficiency (104 cases) and immunosuppressive therapy (70 cases), also showed significant associations.

**TABLE 4:** Top 15 drug indications linked to hyperglycemia/diabetes in children.

Indications	Total	0–4 year	5–9 year	10–14 year	15–18 year
Acute lymphocytic leukemia	192	11	46	74	61
Bipolar disorder	111	3	16	59	33
Growth hormone deficiency	104	1	21	52	30
Attention deficit hyperactivity disorder	83	3	29	38	13
Autism spectrum disorder	71	1	19	25	26
Immunosuppressant drug therapy	70	2	8	13	47
Depression	69	2	6	23	38
Schizophrenia	52	2	5	17	28
Prophylaxis against graft vs. host disease	45	3	5	16	21
Nephrotic syndrome	44	2	4	28	10
Renal transplant	44	1	2	6	35
Affective disorder	41	2	8	9	22
Immunosuppression	36	2	5	15	14
Anxiety	34	0	5	9	20
Asthma	32	9	4	9	10

Our age-stratified analysis revealed distinct patterns across pediatric groups. In the 0–4 year cohort, acute lymphoblastic leukemia (ALL, 11 cases) and asthma (9 cases) were predominan. The 5–9 year group showed peak Attention deficit hyperactivity disorder-related cases (29 cases) alongside persistent ALL predominance (46 cases), consistent with typical diagnostic patterns. A notable “dual burden” emerged in 10–14 year-olds, combining sustained ALL prevalence (74 cases) with sharply increasing mental health cases (bipolar disorder 59 cases, depression 23 cases), while nephrotic syndrome (28 cases) represented the third most common indication. The 15–18 year group demonstrated a distinct profile shift toward transplant-related indications (renal transplant 35 cases, GVHD prophylaxis 21 cases), with depression (38 cases) surpassing bipolar disorder (33 cases) as the primary psychiatric association, coinciding with peak immunosuppressive therapy cases (47 cases).

### 3.5 Medication duration of drug- hyperglycemia/diabetes

The median time from drug initiation to the onset of hyperglycemia/diabetes varied substantially across age groups (Table 5). The 0–4-year group had the shortest median onset at 8 (IQR 3–93) days; the 5–9-year group extended to 149 (32–319) days; the 10–14-year group advanced to 60 (30–317) days; and the 15–18-year group again showed a marked delay, with a median of 160 (60–346) days.

**TABLE 5:** Time-to-onset profiles of drug-induced hyperglycemia/diabetes by age group.

Age group (year)	Median (IQR), days
0–4	8(3–93)
5–9	149(32.5–319)
10–14	60(30–317)
15–18	160 (60.3–345.8)

## 4 Discussion

Leveraging the FDA Adverse Event Reporting System (FAERS), this investigation constitutes the first systematic delineation of the pediatric DIDM risk-drug repertoire and its age-stratified heterogeneity, thereby furnishing pivotal evidence for clinical pharmacovigilance and regulatory governance.

This study included a total of 2,436 pediatric and adolescent cases of drug-induced diabetes mellitus (DIDM). Epidemiological analysis revealed a distinct bimodal distribution in the age of onset, with the highest incidence rate observed in the 10–14 age group (37.03%), followed by the 15–18 group (34.98%), The median age of patients in this study was 13 years (IQR 9–16), which aligns with the age characteristics (reported as mean ± SD: 14.1 ± 2.1 years) of the study population in the systematic review and meta-analysis by Galling et al. on antipsychotic exposure and type 2 diabetes risk in youth (14). The observed phenomenon may be explained by synergistic β-cell damage caused by pubertal hypersensitivity to insulin resistance combined with concurrent immunosuppressant-glucocorticoid treatment (15). The median onset time exhibited a non-linear age-dependent trajectory: shortest in the 0–4-year cohort (8 days; IQR 3–93), prolonged in the 5–9-year group (149 days; IQR 32–319), shortened again in the 10–14-year cohort (60 days; IQR 30–317), and markedly extended in late adolescence (15–18 years: 160 days; IQR 60–346), coinciding with the greatest inter-individual dispersion.

This study identified acute lymphoblastic leukemia (ALL) as the leading underlying diagnosis in children with DIH/DIDM (192 cases), with the 10–14-year age group representing the largest proportion (38.54%). When the time-to-onset is taken into account, these cases appear to reflect cumulative exposure to prolonged, multi-agent chemotherapy. Scrutiny of the fifteen most frequently implicated agents revealed that vincristine, methotrexate, pegaspargase, cyclophosphamide and cytarabine are all integral to contemporary ALL regimens. Of these, pegaspargase (16, 17), cyclophosphamide (18) and cytarabine (19) carry explicit hyperglycemia warnings in both product labels and published literature, whereas vincristine and methotrexate do not. Induction chemotherapy for ALL routinely incorporates corticosteroids, increasing the risk of hyperglycemia 2.2-fold even in patients without pre-existing diabetes (20). A prospective cohort study reported that 11% of children with ALL developed transient hyperglycemia during induction therapy, with older age and higher BMI conferring additive risk. Notably, all patients who required insulin belonged to the 13–18-year age group and had a positive family history of diabetes; those classified as obese (BMI ≥ 95th percentile) were three times more likely to need insulin than their normal-weight counterparts. These findings underscore that the interaction between adolescent hormonal milieu and increased metabolic load amplifies the severity of chemotherapy-induced hyperglycemia in ALL (20). Pegaspargase depletes circulating asparagine, thereby suppressing insulin synthesis and triggering endoplasmic-reticulum stress and apoptosis in pancreatic β-cells, ultimately resulting in hyperglycemia (21, 22).

Moreover, glucocorticoids rank among the leading causes of DIH/DIDM in children, with the strongest signals observed for prednisone (238 cases; ROR 5.12; 95% CI: 4.48–5.86), dexamethasone (232 cases; ROR 4.72; 95% CI: 4.12–5.41), prednisolone (173 cases; ROR 4.62; 95% CI: 3.96–5.40) and methylprednisolone (158 cases; ROR 4.28; 95% CI: 3.64–5.04). In the pediatric population, these agents are widely prescribed for asthma, hematological malignancies and post-transplant immunosuppression, and accumulating evidence confirms that oral glucocorticoid exposure is significantly associated with incident hyperglycemic events (23). Beyond the ALL setting, two lines of evidence have converged on a tripartite glucocorticoid (GC) diabetogenic axis. Tamez-Pérez et al. demonstrated that supra-physiological GC exposure transcriptionally activates the hepatic FoxO1–PEPCK/G6 Pase pathway, amplifying hepatic glucose output, while simultaneously suppressing IRS-1/PI3K–AKT signaling in skeletal muscle, thereby curtailing GLUT-4 translocation and peripheral glucose uptake (24). Barker et al. extended this paradigm by showing that GCs, via GRα, down-regulate insulin-receptor-substrate expression and trigger β-cell apoptosis, yielding a “liver-centric hyper-production, peripheral insulin resistance, and β-cell failure” phenotype that underlies GC-induced hyperglycemia and diabetes (25).

After grouping related indications, neuropsychiatric disorders totaled 461 cases—substantially surpassing leukemia—and included bipolar disorder (111 cases), Attention deficit hyperactivity disorder (ADHD, 83 cases), autism spectrum disorder (71 cases), depression (69 cases), schizophrenia (52 cases), affective disorder (41 cases) and anxiety disorders. Age distribution rose progressively: 13 cases aged 0–4 years, 88 aged 5–9 years, and a peak of 180 aged 10–18 years. Among the top 15 suspected DIDM drugs, quetiapine, risperidone and olanzapine are all psychotropic agents. A meta-analysis of 185,105 youths aged 2–24 years (comprising 13 studies) found that after ≥ 3 months of antipsychotic treatment, the cumulative incidence of type 2 diabetes was 5.72 per 1,000 individuals, with an incidence rate of 3.09 per 1,000 person-years. Compared with healthy controls, the cumulative risk increased by 2.6-fold (OR = 2.58), and the incidence rate tripled (IRR = 3.02); the risk was also significantly higher than in psychiatric controls not receiving antipsychotics. Longer follow-up duration, olanzapine use, and second-generation antipsychotics further elevated the risk (14). A prospective study revealed that 14.3% of adolescent psychiatric patients developed elevated blood glucose following treatment with atypical antipsychotics, with the highest risk observed in the olanzapine and clozapine groups, while aripiprazole and ziprasidone were associated with relatively lower risks (26) Antipsychotic drugs elicit diabetes via a dual-hit mechanism combining peripheral insulin resistance and β-cell cytotoxicity; direct β-cell apoptosis represents the distinguishing hallmark from high-fat-diet models and mandates a multi-target, integrated strategy for primary prevention (27).

Immunosuppression-related disorders accounted for a notable proportion—239 cases in total—including immunosuppressant drug therapy (70 cases), prophylaxis against graft versus host disease (45 cases), nephrotic syndrome (44 cases), renal transplant (44 cases), and immunosuppression (36 cases). The age distribution peaked at 15–18 years (127 cases). Tacrolimus was the primary suspected agent (265 cases; ROR 4.86; 95% CI 4.28–5.52). Salah et al. (28) followed 21 children aged 2–18 years after first renal transplant for 1 year and reported new-onset diabetes in 7 cases (33.3%), all occurring at a median of 7.8 weeks post-surgery; multivariate analysis identified tacrolimus trough levels > 12 ng/mL as an independent risk factor (OR 4.7, 95% CI 1.3–17.4). A retrospective cohort study enrolled 76 pediatric (<18 years) renal transplant recipients with a median follow-up of 6.1 years; post-transplant diabetes mellitus was confirmed in five patients (6.6%) and impaired glucose tolerance in 7 (9.2%). Cox regression showed that each 1 mg/kg increase in cumulative tacrolimus dose raised PTDM risk 1.8-fold (HR 1.83, 95% CI 1.12–3.01) (29). Emerging evidence indicates that tacrolimus disrupts glucose homeostasis by inhibiting calcineurin, down-regulating hepatic and peripheral insulin signaling pathways, inflicting direct β-cell damage, and perturbing the gut–liver axis, thereby engendering concurrent insulin resistance and secretory failure; notably, a rapid-metabolizer phenotype partially attenuates this diabetogenic toxicity (30–32).

Growth hormone (GH) deficiency is also a common indication associated with DIH/DIDM. The peak incidence occurs between 10 and 14 years of age (52 cases, 50%). The primary suspected drugs are growth hormone preparations, notably somatropin (302 cases; ROR 2.87; 95% CI 2.55–3.23). In a study of 37 children with type 1 diabetes and concomitant GH deficiency, GH therapy increased daily insulin requirements from 0.85 to 1.0 IU/kg (P < 0.01), whereas HbA1c remained unchanged (8.1% vs. 8.2%, *P* > 0.05), indicating that GH raises insulin needs through heightened insulin resistance (33). A large retrospective cohort of over 23,000 children further showed that GH treatment was associated with a six-fold increase in the risk of type 2 diabetes that persisted after therapy cessation, without affecting the incidence of type 1 diabetes (34). Evidence demonstrates that growth hormone, acting as a counter-regulatory hormone to insulin, precipitates insulin resistance by suppressing peripheral glucose uptake, enhancing hepatic gluconeogenesis, and promoting lipolytic release of free fatty acids; therefore, sustained metabolic surveillance is warranted for obese and adolescent populations receiving GH therapy (35, 36).

Leveraging spontaneous-report data from the FAERS database, this study is the first to identify hyperglycemia/diabetes-related safety signals for minocycline, montelukast, lomustine, levetiracetam, carboplatin, and procarbazine in the pediatric population. Brown et al. reported a case of a 15-year-old female who developed drug hypersensitivity syndrome (DHS) following minocycline administration, and subsequently developed type 1 diabetes mellitus 7 months after drug withdrawal. The underlying mechanism may involve minocycline acting as a hapten to bind to host proteins, thereby forming novel antigens. These neoantigens trigger immune activation and cytokine storm, which disrupt immune tolerance, induce the production of islet β-cell-specific autoantibodies, and ultimately lead to islet β-cell damage and insufficient insulin secretion (37). A large number of clinical studies are still needed to verify the effects of these drugs on blood glucose. Reports of pediatric diabetes linked to the remaining agents remain scarce. Despite the absence of label warnings or mechanistic evidence in pediatrics, these off-label associations suggest an expanding pharmacological spectrum of drug-induced hyperglycemia or diabetes mellitus (DIH/DIDM) in children. Given the current lack of causal validation, we recommend integrating these agents into pediatric glucose-monitoring protocols and initiating prospective pharmacovigilance or real-world evidence studies to systematically quantify their causal contribution and underlying mechanisms.

Although this study offers a comprehensive pharmacovigilance-based overview of drugs associated with drug-induced hyperglycemia/diabetes mellitus (DIH/DIDM) in children, several inherent limitations must be acknowledged. First, the reporting odds ratio (ROR) indicates statistical association rather than causation. Moreover, when the reporting odds ratio (ROR) is exceptionally high yet the case count is extremely small (e.g., only 19 reports for lomustine), the pronounced signal is accompanied by substantial sampling error, rendering the effect estimate highly unstable and statistically imprecise. Second, as a spontaneous-reporting database, FAERS is susceptible to selection bias, under-reporting, and duplicate entries, which may either inflate or attenuate the true risk. A non-negligible proportion of records lacked indication data, and the supplied indications were recorded in heterogeneous free-text expressions that precluded uniform mapping. Meanwhile, the majority of reports are based on facts from the United States. Third, residual confounding from sex, age, ethnicity, comorbidities, and concomitant medications was not systematically controlled, thereby constraining causal inference. Consequently, the observed drug–event relationships require validation through prospective cohorts, real-world evidence studies, or randomized controlled trials.

## 5 Conclusion

This study is the first to construct a three-dimensional risk landscape of pediatric DIH/DIDM that integrates age, drug class, and underlying indication. Data demonstrate that the 10–18-year age interval represents a metabolic vulnerability window, with chemotherapeutics, glucocorticoids, immunosuppressants, psychotropic agents, and growth hormone constituting five cardinal risk clusters. Moreover, off-label signals for minocycline, montelukast, lomustine, levetiracetam, carboplatin, procarbazine were captured in the pediatric cohort for the first time, indicating that the pharmacological spectrum of DIH/DIDM continues to expand dynamically. In clinical practice, these high-risk drugs should be incorporated into baseline pediatric glucose-monitoring protocols, with dynamic HbA1c/OGTT surveillance initiated in adolescents and in patients exposed to polypharmacy.

## Data Availability

The raw data supporting the conclusions of this article will be made available by the authors, without undue reservation.
